# A Chemical Theory of Scurvy

**Published:** 1908-07-18

**Authors:** 


### A CHEMICAL THEORY OF SCURVY.

For various reasons, scurvy has nowadays become a rarity. Nevertheless, Ralfe's theory of its pathology, as elaborated by Sir Almroth Wright, possesses practical importance as well as much intrinsic interest, because infantile scurvy, or "scurvy-rickets," which is so closely allied to true scurvy, is unfortunately by no means uncommon. According to these two workers, the disease consists primarily in a diminished alkalinity of the blood, as found by neutralisation of a small quantity *in vitro*. Now it can be shown by a process of exclusion, first, that the faulty diet which produces scurvy is faulty by reason of its deficiency in mineral ingredients; and secondly, that the particular chemical group wanting is that containing those salts whose acids form alkaline carbonates in the system—*i.e.*, lactates, citrates, acetates, tartrates, and malates. The converse part of the proof is supplied by the fact that addition of these particular acids and salts to the diet cures the true scorbutic condition with great certainty. It is conjectured that they are important nutritional agents. At first neutral, they become alkaline from conversion into carbonates, and thus probably help to neutralise the continual excess of acids (phosphoric, uric, hippuric, etc.), produced during nutrition: the only other sources of alkali within the body are ammonia and phosphates, both of which are not likely to act to any extent as ant-acids. As a matter of experiment, a dose of sodium lactate will render the urine alkaline in a few minutes, and by this route possibly excess of alkali is removed, since, according to Wright, it is not possible to raise the alkalinity of the blood above the normal, although by a diet faulty in the respects just noticed it may be lowered. In scurvy, then, the patient's condition is one of acid intoxication. If this be granted in its entirety (and some hesitation is perhaps necessary here), there is great play for some of Sir Almroth Wright's conceptions in the explanation of the well-known signs and symptoms of this disease. First, as to the numerous hæmorrhages. Hæmorrhage is likely to be associated with low hæmo-coagulability, and this Wright has abundantly shown to be a condition often accompanied by deficiency of calcium in the blood. Such deficiency might easily be brought about by chemical decalcification, the result of the action of insufficiently neutralised acids. Certainly there seems in scurvy to be a special tendency to decalcification. Everyone knows of the separation of the bones from the epiphyses common in the infantile form. In "Hakluyt's Voyages" (scurvy has always been "the calamity of sailors") it is stated that in an epidemic of this disease it was noticed that those who had recovered from fractures sustained in rough weather experienced in the early part of a cruise suffered re-separation of the fragments after developing scurvy. The same thing has been recorded in more modern times. Another result of low coagulability, and low lime-content, of the blood—according to Wright—is a tendency to serous exudation. Now, œdema of the ankles and brawny infiltrations of the subcutaneous tissues are features of the disease under notice. The therapeutic indication is obvious—to wit, immediate use of sodium lactate. Estimation of the alkalinity of the blood requres very little technique, and may occasionally be well worth undertaking; for instance, Wright has reported a case of a diabetic on strict diet developing serious symptoms which quickly yielded to hæmo-alkalinisers.

